# Mr. R. G. Paul

**Published:** 1980

**Authors:** 


### Mr. R. G. Paul
### L.R.C.P., L.R.F.P.S., F.R.C.S.

Although it is over ten years since Gordon Paul was engaged in active surgical practice in Bristol, he will be remembered by a large number of both doctors and patients because he led a very busy life both on the staff of the Bristol Royal Infirmary and in private practice. In addition, he was very proud to serve Cossham Hospital and Devizes and District Hospital.

Mr. Paul obtained many national honours. He was a graduate of Edinburgh University in 1927. He became a Fellow of the Royal College of Surgeons in Edinburgh, and was very proud when he was elected Fellow of the Royal College of Surgeons of England (ad eundem) in 1959. In addition, Mr. Paul became a Fellow of the Association of Surgeons of Great Britain, and a member of the council. He was a member and past president of the Moynihan Chirurgical Club, and a member of the Surgical Club of South West England.

It is not only for his academic career that he will be remembered. He was a first class surgeon, and many people owe him a debt of gratitude for his clinical judgment and surgical skill. He took a special interest in the large bowel, and particularly the rectum and anal canal. His spincter preserving operations for carcinoma of the rectum were many years in advance of the general move to the universal adoption of this procedure. His other special surgical interest was in breast carcinoma and here again he was 10 years in advance of the general practice in abandoning Radical Mastectomy for a less mutilating operation preserving pectoralis major muscle, now generally adopted. No one who came to him for help went away without realising that he was a man of deepest sympathy with a great concern for their welfare. Rich or poor, old or young, it made no difference. He gave to each and everyone equal attention, and his caring spirit came through to gain their confidence.

Mr. Paul was a teacher who was highly respected and sought after by both undergraduates and graduates alike. His ward rounds were most popular and his bedside teaching was outstanding. His postgraduate lectures and teaching sessions for trainee surgeons revealed a depth of knowledge which his modesty often belied. His every activity was offset by a wonderful sense of humour which was a great asset and much appreciated by everyone.

Many surgeons will look back with heartfelt gratitude to the time that they spent with Gordon Paul. His technical skill must have encouraged and inspired many, and when they sought his advice it was always freely available.

In his spare time he was a keen sportsman and an expert fisherman. Devon, South Wales and Scotland were areas which he often visited, and his sudden tragic death occurred in Dulverton where he spent many enjoyable holidays both with his wife and with colleagues who were fishing companions.

In his later life he enjoyed the company of fellow members of the Bristol Medical Reading Society. The monthly meetings were a source of strength and fellowship, particularly after the tragic death of his wife in 1977.

Mr. Paul had a very distinguished career in the RAMC in the 1939–45 war, and was consultant surgeon Southern Army India Command with the rank of Brigadier when he was demobilised. He brought great distinction to Bristol, his adopted City and University, and he will be remembered by all of us as a man of unlimited kindness and devotion. Nothing was ever too much trouble, and he is sadly missed by colleagues, friends and grateful patients.

